# Heat tolerance, oxidative stress response tuning and robust gene activation in early-stage *Drosophila melanogaster* embryos

**DOI:** 10.1098/rspb.2024.0973

**Published:** 2024-08-21

**Authors:** Emily E. Mikucki, Thomas S. O’Leary, Brent L. Lockwood

**Affiliations:** ^1^ Department of Biology, University of Vermont, Burlington, VT, USA

**Keywords:** heat shock response, embryogenesis, maternal-to-zygotic transition, transcriptomics, environmental change, thermal adaptation

## Abstract

In organisms with complex life cycles, life stages that are most susceptible to environmental stress may determine species persistence in the face of climate change. Early embryos of *Drosophila melanogaster* are particularly sensitive to acute heat stress, yet tropical embryos have higher heat tolerance than temperate embryos, suggesting adaptive variation in embryonic heat tolerance. We compared transcriptomic responses to heat stress among tropical and temperate embryos to elucidate the gene regulatory basis of divergence in embryonic heat tolerance. The transcriptomes of tropical and temperate embryos differed in both constitutive and heat-stress-induced responses of the expression of relatively few genes, including genes involved in oxidative stress. Most of the transcriptomic response to heat stress was shared among all embryos. Embryos shifted the expression of thousands of genes, including increases in the expression of heat shock genes, suggesting robust zygotic gene activation and demonstrating that, contrary to previous reports, early embryos are not transcriptionally silent. The involvement of oxidative stress genes corroborates recent reports on the critical role of redox homeostasis in coordinating developmental transitions. By characterizing adaptive variation in the transcriptomic basis of embryonic heat tolerance, this study is a novel contribution to the literature on developmental physiology and developmental genetics.

## 1. Introduction

All life is inherently sensitive to sudden changes in temperature due to the disruption of the weak bonds that stabilize macromolecular structures [1]. Survival against sudden, acute increases in temperature, such as would be encountered during a heat wave or fever, necessitates cellular coping strategies, like the heat shock response, that aid cells in mitigating macromolecular damage [2–6]. The heat shock response is a fast transcriptional response characterized by the differential regulation of thousands of genes [7–9]. While other aspects of thermal physiology influence upper thermal limits, such as lipid membrane composition [10], the heat shock response directly confers whole-organism heat tolerance [11–13]. Consequently, variation in transcriptional regulation in response to heat stress has been the target of thermal selection in many species [14–17] and may be critical to surviving climate warming [6,18], as the global climate crisis is leading to increases in mean temperatures and the frequency of heat waves [19,20].

The reliance on transcriptional regulation for heat stress tolerance poses physiological constraints that may limit adaptive responses to heat waves. For example, the Antarctic fish *Trematomus bernacchii* lacks the ability to induce the expression of heat shock proteins [21] and dies of heat stress at 6°C [22]. In species with complex life cycles, the life stage with the greatest degree of sensitivity to heat stress will most likely set its upper thermal limits [23,24]. In the context of the heat shock response, the earliest stages of embryogenesis may be particularly thermally sensitive, as early embryos in all animals and endosperms in higher plants lack a fully developed transcriptional machinery [25,26]. But despite the potential constraints to heat tolerance in early life stages, previous work has demonstrated genetic variation in heat tolerance in early life stages that correlates with variation in the thermal environment [24,27,28], suggesting that thermal adaptation is possible even when there appear to be physiological constraints to coping with heat stress in early life stages.

Because early life stages are often thermally sensitive, they are likely to be critical to the thermal ecology of many species [29]. Yet, early life stages are rarely studied in ecologically relevant thermal contexts [24,27,30]. Embryos of *Drosophila melanogaster* are immobile, and thus at the mercy of the environment where their mothers lay them. Within hours, the temperature of a piece of necrotic fruit, the preferred oviposition site for *D. melanogaster*, may increase by as much as 20°C [31]. Unlike adult flies, embryos cannot avoid thermal extremes through behavioural thermoregulation [32–34]. Moreover, early-stage (0- to 3-hour-old) *D. melanogaster* embryos are more thermally sensitive than all other life stages [13,24,35,36]. While all of embryogenesis is predicted to be sensitive to heat stress due to potential disruptions to mitosis that lead to developmental arrest [37,38], early-stage embryos are uniquely more thermally sensitive than later stages of embryogenesis, leading some to suggest that the thermal sensitivity of early embryos stems from their reduced transcriptional activity [13,39,40]. However, the extent to which maternal provisioning of mRNAs may protect early embryos against heat stress has not been fully explored (but see [40]). Furthermore, natural variation in the transcriptome of early embryos of *D. melanogaster* has not been previously measured, and transcriptomic responses to acute heat stress in early embryos have never been characterized.

Here, we characterized the transcriptomes of tropical and temperate *D. melanogaster* early embryos in the context of acute heat stress to elucidate potential molecular physiological bases of adaptive thermal tolerance. *D. melanogaster* has a broad global distribution [41], and previously, we demonstrated that tropical *D. melanogaster*, collected from relatively hot locations around the globe, produce embryos that are more heat tolerant than embryos of temperate flies collected from relatively cool locations in North America [24]. Fundamentally, the physiology of early embryogenesis is controlled by gene activation [42–44], and thus we sought to characterize transcriptomic responses to elucidate the potential mechanisms that underlie this adaptive divergence.

Embryonic heat tolerance could manifest in several ways at the level of the transcriptome. First, tropical embryos may constitutively express genes involved in heat stress coping mechanisms in preparation for heat stress [45,46]. Second, tropical embryos may respond to heat stress by inducing the expression of heat stress genes [7–9,16]. Or, third, tropical embryos may have an accelerated development rate and be more transcriptionally active overall due to an earlier onset of gene activation. Our results allow us to address these possibilities to determine how the transcriptome mediates responses to environmental variability in a developmental context. In addition, to our knowledge, this is the first study to compare the early-life transcriptomes of broadly sampled *D. melanogaster* populations around the globe.

## 2. Material and methods

### (a) Fly strains

**Figure 1.**
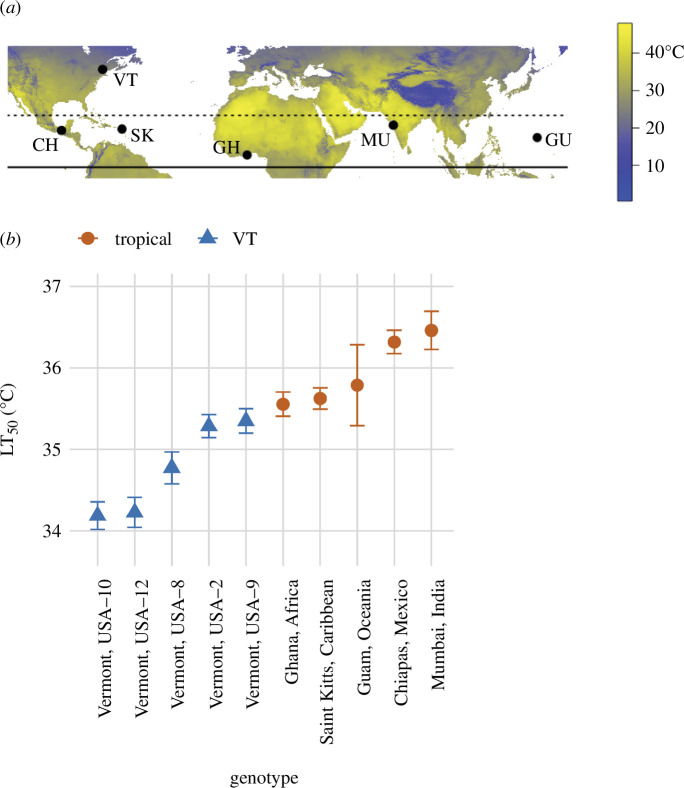
(*a*) Collection locations: VT = Vermont, USA, CH = Chiapas, Mexico, SK = Saint Kitts, Caribbean, GH = Ghana, Africa, MU = Mumbai, India and GU = Guam, Oceania. Colour scale indicates the average maximum temperature of the warmest month of the year (1970–2000, Worldclim) [47]. (*b*) *D. melanogaster* embryonic heat tolerance as indicated by LT_50_—the temperature that causes 50% mortality following a 45 min exposure—among the ten genotypes used in this study. For details, see Lockwood *et al*. [24].

We propagated five tropical and five temperate North American iso-female lines of *D. melanogaster* as described previously [24]. These lines exhibit continuous variation in embryonic heat tolerance, as indexed by survival (LT_50_) following acute heat stress (45 min), with the tropical lines having the highest heat tolerance (figure 1). Tropical lines were obtained from the Drosophila Species Stock Center with origins from Mumbai, India (MU) (Stock number: 14021-0231.45), Accra, Ghana, Africa (GH) (Stock number: 14021-0231.182), Monkey Hill, St Kitts, Caribbean (SK) (Stock number: 14021-0231.34), Chiapas de Corzo, Chiapas, Mexico (CH) (Stock number: 14021-0231.22) and Guam, Oceania (GU) (Stock number: 14021-0231.198). Vermont lines were collected from East Calais, VT [48]. Iso-female lines were established from single females whose progeny were inbred for several generations to isogenize the genetic background. This minimizes laboratory evolution by removing genetic variability [48]. We maintained flies at densities of 50–100 adults per vial (95 × 25 mm^2^) under common-garden conditions in incubators (DR-36VL, Percival Scientific Inc., Perry, Iowa, USA) at 25°C, 55% humidity, and a 12L : 12D light cycle on a cornmeal–yeast–molasses medium for several generations prior to collecting embryos for heat shock. In the wild, 25°C is an ecologically relevant temperature that tropical and North American *D*. *melanogaster* flies experience.

### (b) Embryonic heat shock

Heat shocks mimicked the natural, acute increases in temperature of the necrotic fruit on which *D. melanogaster* oviposit their eggs [31], spanned the range of LT_50_ values of the ten iso-female lines (figure 1) and mimicked the design of the original study of the LT_50_ phenotype [24]. We collected eggs at 0–1 h post-fertilization from three- to five-day-old adult flies (n ≈ 100 pairs) on grape juice agar plates (60 × 15 mm^2^) dabbed with active baker’s yeast at 25°C. To control for embryo stage, we purged embryos that may have been previously fertilized by collecting eggs for 1 h and discarding them [49]. We then provided fresh plates for egg collection. Thus, embryos were between 0 and 1 h post-fertilization at the start of the temperature treatments. We then wrapped the egg plates with parafilm and submerged them in a circulating water bath (A24B, Thermo Scientific, Waltham, MA, USA) for 45 min at one of four temperatures: 25°C, 32°C, 34°C and 36°C. The plates increased in temperature at a rate of +0.4°C min^−1^ until reaching the target temperature [24]. We repeated the heat shocks for a minimum of three replicates per treatment (genotype × temperature).

After heat shock, we rinsed eggs with 0.12 M NaCl and 0.05% (v/v) Triton X-100 for 30 s to remove residual yeast. We then dechorionated embryos by washing them with 50% bleach for 1 min and rinsing in dH_2_O for 30 s. Removal of the chorion facilitates homogenization [50], and short exposure to 50% bleach does not disrupt development [49] or induce cellular stress responses [51]. We removed residual water by blotting with a Kimwipe. We pooled eggs (*n *≥ 30) from each plate into 1.5 ml microcentrifuge tubes and immediately flash froze them in liquid nitrogen. Embryos were preserved at −80°C until RNA extraction.

### (c) RNA extraction and sequencing

We extracted total RNA by homogenizing embryos in 250 µl of TRIzol (Tri-reagent, Sigma Life Science, St Louis, MO, USA) and centrifuging at 4°C at 6000 × *g* for 1 min (Sorvall ST89, Thermo Scientific). We then added 150 µl of TRIzol and 150 µl of chloroform (Sigma), followed by centrifugation at 4°C at 12 000 × *g* for 10 min (Sorvall ST89, Thermo Scientific). We precipitated each sample with isopropanol and ethanol. We determined RNA quantity with a Nanodrop spectrophotometer (NanoDrop 2000, Thermo Scientific). We assessed RNA quality on agarose (2%) gels (EL-200, Walter, Plymouth, MI, USA). We sequenced samples containing >1 µg of RNA and that exhibited intact 28S and 18S ribosomal RNAs bands.

We sent all samples (*n* = 120) to Novogene (Sacramento, CA, USA) for 150 base-pair paired-end mRNA sequencing. Novogene performed quality control with a Nanodrop, agarose gel electrophoresis and an Agilent 2100 BioAnalyzer (Santa Clara, CA, USA). A total of 110 of the 120 samples passed the second round of quality control. Ten samples had signs of RNA degradation (RIN < 3.0) and thus were not sequenced. The excluded samples were: MU at 34°C, MU at 36°C, CH at 36°C, GH at 32°C, GU at 32°C, VT12 at 25°C, VT2 at 32°C, VT2 at 36°C (×2) and VT9 at 25°C.

Sequencing libraries were prepared with a TruSeq RNA Library Prep Kit v2 (Illumina, San Diego, CA, USA), enriching for mRNA with oligo(dT) beads and synthesizing cDNA with random hexamer primers. Double-stranded cDNA libraries were sequenced on a Novaseq 6000 (S4 Flowcell) (Illumina, San Diego, CA, USA). Library quality was assessed using a Qubit 2.0 fluorometer (Thermo Scientific), Agilent 2100 BioAnalyzer and q-PCR.

The RNA sequencing yielded a total of 1.32 billion reads with 6.0 million ± 0.75 million (mean ± s.d.) paired-end reads per sample. We checked the quality of raw sequence reads using FastQC (v. 0.11.5) [52]. We trimmed the forward and reverse reads using Trimmomatic (v. 0.36) [53]. While adapter sequences may not interfere with the mapping of RNAseq reads [54], we followed Illumina’s recommendations for best practices and trimmed adapter sequences prior to mapping (Illumina; https://knowledge.illumina.com/software/general/software-general-reference_material-list/000002905, accessed 2024). Trimmomatic parameters were set for paired-end data with Phred 33 encoding to remove adapter sequences and trim low-quality (Phred < 20) bases of the first 20 bases and the 20 trailing bases of each read. We also used a 6-base sliding window to trim low-quality bases (Phred < 20). We dropped reads shorter than 35 bases.

### (d) Quantification, normalization and differential expression analysis

We used salmon (v. 1.1.0) to map transcripts to the *D. melanogaster* transcriptome (FlyBase v. r6.34) [55]. There was an average of 5.4 million ± 0.7 million (median ± s.d.) paired-end fragments that were mapped as counts per sample. This degree of coverage is similar to that of recently published transcriptomics studies of early *D. melanogaster* embryos [56]. Further, our differential expression analysis was bolstered by a high level of replication (*n* = 13–15 per experimental group), which has been shown to be a more important factor than sequencing depth for detecting differentially expressed genes, even for lowly expressed genes [57]. There was an average of 14.6 thousand ± 1.5 thousand (median ± s.d.) transcripts detected per sample. Statistical analyses were conducted at the level of transcripts, and we report the number of differentially expressed transcripts and genes in the Results (electronic supplementary material, figure S3). We used R (v. 4.2.3) [58] and the package DESeq2 (v. 1.32.0) to normalize read counts and conduct differential expression analysis [59]. We conducted the likelihood ratio test (LRT) with the DESeq function, treating region of origin and temperature as factors, to determine transcripts with significantly different abundances based on region, temperature and region × temperature interactions (FDR < 0.05). To match transcripts to genes, we used the R package gprofiler2 [60]. We conducted principal component analysis using the prcomp function in R.

### (e) Co-expression analysis

To identify modules of co-expressed transcripts that correlate with LT_50_ or temperature, we performed weighted gene co-expression network analysis (WGCNA) with the WGCNA package in R [61]. Following the recommendation of the authors of WGCNA, we filtered out transcripts with fewer than 15 counts in more than 75% of the samples. This rendered 8754 transcripts, which we used to construct the network. We performed variance stabilization to normalize the counts of the filtered dataset with the vst function in DESeq2. We then constructed a signed network with a soft-power threshold of 10, which provided a good model fit (*R* ^2^ = 0.91; electronic supplementary material, figure S1) while maintaining a high degree of connectivity (mean connectivity = 161.6). We used a merge cut height of 0.25, which merged modules that were 75% similar in their clusters of transcripts. These parameters produced 17 modules of co-expressed transcripts. The module expression is the principal component (PC1) of gene expression in each module and represents a summary of expression profiles of co-expressed transcripts in the module. With signed networks, positive correlations between module expression values and trait values (i.e. LT_50_) indicate a higher abundance of transcripts corresponds to higher trait values [61,62].

### (f) Functional enrichment analysis

We conducted functional enrichment analysis with the R package VISEAGO [63]. We focused our analyses on biological process categories and ran the classic algorithm of overrepresentation analysis using the Fisher’s exact test with an FDR of 0.01. The broad functional categories shown in electronic supplementary material, figure S5*b*, are not GO terms and were curated by the authors to present a broad classification of the cellular and developmental processes in the study system. We used FlyBase (release FB2023_06) [64], along with the published literature, to classify gene function.

## 3. Results

### (a) Transcriptomic correlates of embryonic heat tolerance

Overall, embryonic heat tolerance (figure 1*b*) was associated with the expression of relatively few genes, as transcriptomes were similar regardless of region of origin (electronic supplementary material, figure S2). A total of 793 genes were differentially expressed between tropical and VT embryos (electronic supplementary material, figure S3; DESeq2, LRT, region factor, FDR < 0.05), with 285 of these genes also showing significant changes in expression following heat stress (electronic supplementary material, figure S3). But no genes showed significant region-specific responses to temperature (electronic supplementary material, figure S3; DESeq2, LRT, region × temperature interaction, FDR > 0.05), at least based on analysis with DESeq2 (but see below). The 793 genes with differences in transcript abundance between regions were encoded by 799 mRNA transcripts, and most of these were more highly expressed in tropical embryos than VT embryos—429 transcripts were more abundant in tropical versus 370 transcripts that were more abundant in VT embryos. Overall, 3826 genes corresponding to 4135 transcripts showed significant changes in expression following heat stress (electronic supplementary material, figure S3; DESeq2, LRT, temperature factor, FDR < 0.05), indicating a robust heat shock response that was shared among all embryos (see §3b).

**Figure 2.**
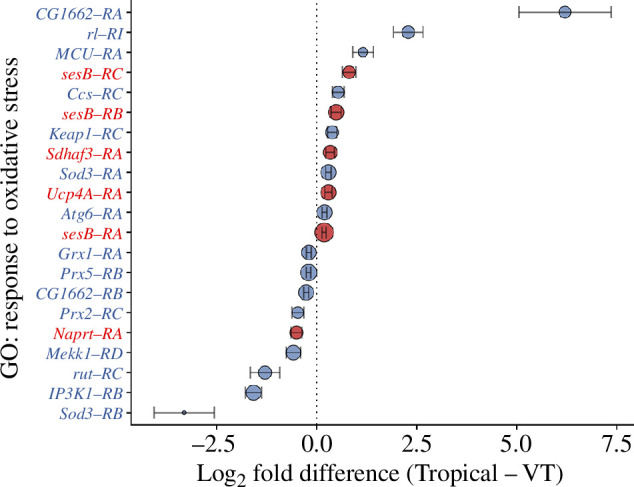
Mean differences in expression of oxidative stress response transcripts between tropical and Vermont embryos across all temperatures. Positive numbers indicate higher expression in tropical embryos. Negative numbers indicate higher expression in Vermont embryos. Transcripts in blue are involved in general resistance to oxidative stress, either by direct scavenging or by a cellular response to ROS. Transcripts in red are involved in the prevention of ROS formation. The size of the points indicates the mean normalized expression (log_2_ scale). Error bars indicate standard error of the mean.

There was little functional overlap between the genes that were differentially expressed between tropical and VT embryos and the heat stress response genes (electronic supplementary material, figure S4*a*, table S1). A total of 72 GO function categories distinguished tropical and VT transcriptomes (electronic supplementary material, figure S4*a*), and nine of these were associated with the oxidative stress response (electronic supplementary material, figure S4*b*). Of note, many GO categories associated with cellular stress were enriched among the genes that changed expression upon heat stress, but only the oxidative stress response was different between tropical and VT embryos (electronic supplementary material, figure S4*b*). Among the 17 genes in the ‘response to oxidative stress’ GO category, eight were more highly expressed in tropical embryos and seven were more highly expressed in VT embryos, with two genes having two transcript isoforms each that were more highly expressed in either tropical or VT embryos (figure 2). The specific functional roles of these genes could be classified as contributing to either the prevention of the formation of reactive oxygen species (ROS) or resistance to oxidative stress (electronic supplementary material, table S2). Notably, four of the 17 genes were involved in ROS prevention, and three of these were more highly expressed in tropical embryos (electronic supplementary material, table S2; figure 2).

**Figure 3.**
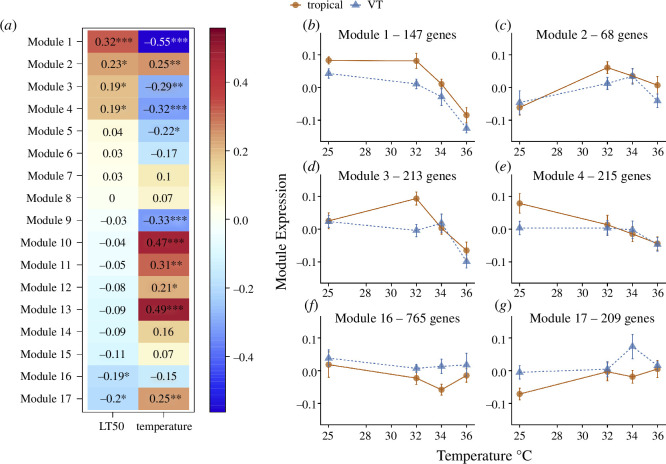
Modules of gene expression identified by WGCNA. (*a*) Pearson correlation coefficients between module expression and LT_50_ or temperature. Red colours indicate positive correlations, and blue colours indicate negative correlations. Asterisks indicate a level of significance of the Pearson correlation: *p < 0.05, **p < 0.01 and ***p < 0.001. (*b–g*) Module expression values among transcriptomes of a given region (mean ± s.e.m.) and across temperature for the six modules with significant correlations to LT_50_. Note that module expression reflects the pattern of expression common to all genes in a particular module, with higher values indicating greater abundance of RNA.

We identified six modules of gene expression, representing a total of 1617 genes, whose expression was correlated with LT_50_ (figures 1 and 3; electronic supplementary material, table S3; WGCNA). Notably, these modules also distinguished transcriptional responses between tropical and VT embryos (figure 3*b–g*). Four out of the six modules had expression profiles that were positively correlated with LT_50_ (figure 3*a*). For example, Module 1 had expression values that were higher in tropical embryos across all temperatures, indicating differences in constitutive expression (figure 3*b*), whereas Modules 2 and 3 showed higher expression in tropical embryos at 32°C, indicating differences in response to heat stress (figure 3*c,d*). On the other hand, Modules 16 and 17 had expression values that were negatively correlated with LT_50_, and thus contained genes whose expression was higher in VT than tropical embryos (figure 3*a,f,g*). These two modules highlight differential responses between tropical and VT embryos to 34°C; Module 16 showed lower expression at 34°C only in tropical embryos, whereas Module 17 showed higher expression at 34°C in VT embryos. Overall, the gene expression patterns most correlated with higher heat tolerance were characterized by higher expression in tropical embryos, either at baseline (25°C) or at the lowest heat shock temperature (32°C), suggesting that preparation for heat shock and the initial response to heat shock were transcriptomic signatures of embryonic heat tolerance.

As stated above, we did not detect any genes with region-specific responses to temperature via DESeq2 analysis. Yet, WGCNA identified modules (i.e. Modules 2, 3, 4, 5 and 6) with expression values that differed by region at one temperature, resembling an interaction between region and temperature (figure 3). We interpret this discrepancy to be the result of (i) WGCNA inherently describing patterns of hundreds of co-expressed genes, which may highlight relatively subtle patterns of average expression that are missed in the gene-by-gene analysis of DESeq2, and (ii) DESeq2 performing false-discovery rate correction, a process that is absent from WGCNA analysis, and which may increase type II error (false negative) rate. Indeed, interaction terms in factorial designs have fewer degrees of freedom than main effects and thus may suffer from lower statistical power [65]. Nonetheless, we include the results of both sets of analyses to highlight different aspects of the data, with DESeq2 highlighting the individual genes with greatest contrasts between regions and WGCNA identifying broadscale patterns of co-expression that correlate with the trait of interest (i.e. heat tolerance).

Functional enrichment analysis of the six modules correlated with LT_50_ (figure 3) revealed a diverse molecular signature of embryonic heat tolerance (electronic supplementary material, figure S5). Overall, each module consisted of genes involved in GO categories that were distinct from other modules (electronic supplementary material, figure S5*a*). However, GO categories could be grouped into higher-order functional families that were shared among multiple modules (electronic supplementary material, figure S5*b*; see §2f for a description of functional annotation). Among the modules positively correlated with heat tolerance (figure 3), Modules 1 and 3 included many genes spread across a wide array of functional categories, whereas Module 2 consisted of genes in only certain categories, including RNA processing, transcription, protein and lipid metabolism and regulatory pathways. Similarly, Module 4 had a relatively specific GO signature, with genes involved in protein and lipid metabolism, morphogenesis, localization and transport and organellar organization. The two modules negatively correlated with heat tolerance, Modules 16 and 17 (figure 3), had a predominance of genes involved in RNA processing, transcription and protein and lipid metabolism. Interestingly, Module 17 had many genes involved in histone modification, indicating a higher order mechanism for differential transcriptional regulation between heat-tolerant and heat-sensitive embryos.

### (b) The embryonic transcriptomic response to heat stress

**Figure 4.**
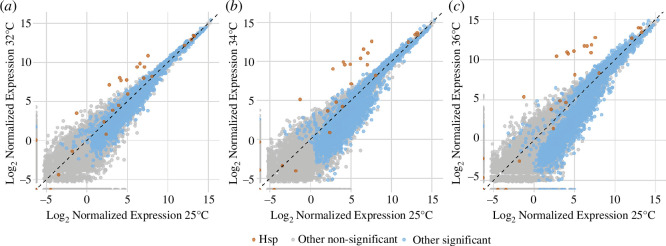
The shared embryonic heat shock response. Plotted are mean log_2_-normalized expression at 25°C versus 32°C (*a*), 34°C (*b*) or 36°C (*c*). Red dots are heat-shock-protein genes (Hsp), grey dots are non-significant non-heat-shock-protein genes, and blue dots are significant non-heat-shock-protein genes (FDR < 0.05). The diagonal dashed line is the 1:1 line of the *x* and *y* axes.

As mentioned above, the transcriptomes of both tropical and temperate embryos were responsive to temperature, with thousands of transcripts changing abundance following heat stress (figure 4). Most of the transcripts (2921) decreased in abundance following heat stress, while 1214 transcripts increased in abundance (figure 4). Based on WGCNA clustering, we identified six modules, representing 2772 genes, with module expression significantly correlated to temperature and not heat tolerance (figure 3*a* and electronic supplementary material, figure S6). Thus, these modules represent the transcriptional response to heat stress that all embryos shared, with some modules decreasing and others increasing in expression. GO analysis revealed a diverse array of functional categories that were highly enriched in these modules (electronic supplementary material, table S4). Notably, the modules that decreased in expression (i.e. Modules 5 and 9) contained genes involved in translation (GO:0006412) and peptide biosynthesis (GO:0043043). Whereas the modules that increased in expression (i.e. Modules 10, 11, 12 and 13) contained genes involved in cellular localization (GO:0051641), cellular component organization (GO:0016043) and the cellular response to stress (GO:0033554). Molecular chaperone activity was evident in Module 13, with protein refolding (GO:0042026), chaperone-mediated protein folding (GO:0061077), and heat-shock mediated polytene chromosome puffing (GO:0035080) being among the top five most enriched categories (electronic supplementary material, table S4). Indeed, Module 13 contained many of the classic heat shock genes (e.g. Hsp70 Bb, Hsp68 and Hsp23), all of which were highly induced following heat stress (figure 4 and electronic supplementary material, figure S6).

## 4. Discussion

### (a) Expression of few genes underlies embryonic heat tolerance

Our data suggest that the expression of relatively few genes differentiates heat-tolerant from heat-sensitive embryos in *D. melanogaster*. A potential explanation for the similar transcriptomes is the highly conserved nature of embryogenesis [66], such that embryonic transcriptomes are canalized [67,68]. Indeed, gene expression in early development is evolutionarily conserved within *D. melanogaster* and across the *Drosophila* genus [69]. But despite transcriptomic similarities, thermal adaptation of heat tolerance is not constrained, as tropical and temperate embryos have different heat tolerances (figure 1) [24]. Previous studies have shown that transcriptomic responses to heat stress are similar among differentially thermally adapted organisms, but differences in the expression of a few key genes underlie heat tolerance [16,70]. In adults of *D. melanogaster* and *Drosophila simulans*, populations from Maine and Panama exhibited transcriptomic divergence in responses to more benign temperatures of 21°C and 29°C [71]. But, here too, most genes were not differentially expressed between populations. Our data demonstrate that *Drosophila* embryos are consistent with this overall pattern.

An alternative explanation for the relatively minor variation among transcriptomes is that thermal selection acts on levels of biological organization other than the transcriptome. For example, the evolution of thermal stability of proteins [72–74] may influence the temperature at which the cellular stress response is induced [75]. Thermal stabilities of dehydrogenase enzymes differ among populations of *D. melanogaster* [76], but the data are mixed depending on the enzyme [77,78]. Most examples of thermal adaptation of protein stability are from divergent species and not populations within a species [1,74]. Thus, it is unclear if the evolutionary timescales that separate divergent populations of a species are long enough to render this as a plausible explanation for the data we report on herein. Nonetheless, we cannot rule out the possibility of thermal adaptation via other levels of biological organization that we did not characterize. In fact, our data suggest a potential role for histone post-translational modifications in embryonic heat tolerance. But we also emphasize the likelihood that thermal selection acts on the transcriptome, as demonstrated in fish, lizards, *Daphnia* and copepods [17,27,79–81]. Given the out-sized role of gene activation in coordinating early developmental events [44,82], we believe that transcriptional regulation is likely to be critical to any developmental phenotype, including embryonic heat tolerance.

Based on the patterns of differential expression between tropical and temperate embryos, both preparedness (i.e. constitutive expression) and responsiveness of the transcriptome likely underlie embryonic heat tolerance. Perhaps the best examples of constitutive expression underlying heat tolerance are in the heat-tolerant intertidal limpet *Lottia scabra* and in laboratory-evolved strains of heat-tolerant *Escherichia coli*, both of which exhibit constitutively high levels of heat shock protein expression [45,46]. In *D. melanogaster*, the inducibility of the heat shock response has been genetically manipulated to increase the heat-inducible expression of HSP70 protein and thereby increase whole-organism heat tolerance [11,13]. In addition, naturally segregating variants of a regulatory polymorphism in the Hsp70 gene in *D. melanogaster* exhibit clinal variation across latitude in eastern Australia [14], suggesting that thermal selection acts on the inducibility of the heat shock response in natural populations.

In terms of physiological mechanisms, genes involved in the oxidative stress response appear to be central to embryonic heat tolerance. Some oxidative stress genes encode proteins that actively scavenge ROS after they are formed, such as *Sod3* [64,83], while others encode proteins that prevent ROS from forming altogether, such as *Ucp4A* [1,64,84]. Both tropical and Vermont embryos expressed oxidative stress genes involved in the scavenging of ROS or otherwise supporting stress resistance. However, tropical embryos were distinguished in their ability to have higher expression of genes involved in the prevention of ROS, such as *Ucp4A*, *sesB* and *Sdhaf3*. Thus, the prevention of oxidative stress may be a key mechanism underlying thermal adaptation in early *Drosophila* embryos.

The link between thermal stress tolerance and oxidative stress has been observed across a broad range of taxa [85–88]. Thermal stress can lead to oxidative stress via increased production of ROS, leading to changes in cellular redox potential [1,89]. While ROS can cause cellular damage by oxidizing DNA, RNA, lipids and/or proteins [90,91], ROS are also important cellular signalling molecules involved in the regulation of homeostatic processes [91–93]. Indeed, recent studies have highlighted the critical role of cellular redox state for the coordination of developmental transitions [94,95], cell fate determination [95] and energy metabolism [96,97] during embryogenesis. Because basal levels of ROS preserve cellular and developmental function, antioxidant systems are important for maintaining the delicate balance of ROS [93] and ensuring that excess ROS do not lead to redox imbalance and cellular dysfunction [91]. Altogether, our results suggest that heat stress produces excess ROS that disrupt embryonic development and that more heat-tolerant embryos are better able to prevent ROS formation and thus maintain redox homeostasis during heat stress.

Beyond the oxidative stress response, a wide array of cellular and developmental processes appears to be involved in producing embryos with higher heat tolerance. Indeed, the expression of hundreds of genes was correlated with LT_50_, including genes involved in RNA processing, histone modification, morphogenesis and ion homeostasis. So, even though a relatively small fraction of genes differentiated the transcriptomes of tropical and Vermont embryos, this small fraction performed a diverse set of functions. Because of the pervasive effects of temperature [1,98] it is not surprising that a diverse set of genes was associated with heat tolerance. Heat-tolerant embryos had higher expression of these genes prior to heat stress or upon experiencing low levels of heat stress. Previous studies have demonstrated that heat-tolerant organisms exhibit a more robust early transcriptional response to acute heat stress than heat-sensitive individuals [11,13,45,99]. But what makes the present study unique is that while the canonical heat shock genes were actively induced in response to heat stress (see below), heat shock gene expression was indistinguishable between tropical and Vermont embryos. Thus, it appears that either thermal adaptation has proceeded via a unique physiological path in fly embryos, or the thermal physiology of embryogenesis is different from other cellular contexts, such that ROS signalling may be a more critical factor than proteostasis. Because ROS signalling mediates various cellular and developmental processes [94,95], these broader transcriptomic patterns of differentiation between tropical and Vermont embryos may have resulted from the differential response to oxidative stress. Making the causal link between oxidative stress and patterns of transcription across development would require targeted manipulation of the expression of oxidative stress genes, which is beyond the scope of the present study. Nonetheless, this would be worthwhile to pinpoint the mechanism(s) underlying embryonic heat tolerance more precisely.

### (b) A robust early embryonic transcriptomic response

To those who study early embryonic development, perhaps the most surprising result of the present study is that early embryos had any sort of transcriptional response to temperature. Under the current paradigm of metazoan early development, this transcriptional response would not be possible because zygotic transcription is presumed to be silent until the maternal-to-zygotic transition (MZT) [26], which has been shown to take place after 2 h post-fertilization in *D. melanogaster* [100–104]. The embryos in the present study were sampled between 45 min and 1 hour and 45 min post-fertilization (0–1 h development at 25°C +45 min temperature treatment), yet thousands of transcripts exhibited changes in abundance following heat stress, indicating that early embryos are more transcriptionally active than previously described. Some reports demonstrate zygotic transcription in *D. melanogaster* before the MZT, at or prior to nuclear cycle 8 or as early as 1 h post-fertilization, but these studies show the zygotic expression of very few genes, as embryos were developed under the benign condition of a constant 25°C [42,105]. In contrast, we demonstrate a widespread transcriptomic response reminiscent of the canonical heat shock response [4,8], which involves shifts in gene expression that mitigate protein unfolding [1,12,89]. Indeed, we observed robust induction of many genes that encode molecular chaperones, including *Hsp70Bb*, *Hsp23*, *Hsp68* and *Hsp90* (figure 4; Module 13 of WGCNA; electronic supplementary material, figure S5). Importantly, most of these genes were highly induced following heat stress, thus demonstrating an active embryonic transcriptional response. We acknowledge that this is not the first observance of heat shock protein expression in early fly embryos [39], but this is the first demonstration of a transcriptomic response of heat shock genes in early embryos.

Two possible explanations account for this widespread early onset of zygotic transcription upon acute heat shock. First, these results could indicate a shift in the MZT due to the positive relationship between temperature and reaction rates [1]. Embryos develop faster in hotter temperatures [106], and we saw increased expression of transcripts involved in developmental processes at hotter temperatures, including pathways in cellular organization and morphogenesis. We also observed that 2921 transcripts decreased in abundance with increasing temperature. Most of the transcripts that decreased in abundance likely represent maternal transcripts that were being degraded [51,69] because they were present at highest abundances at 25°C. The degradation of these maternal transcripts may be an outcome of the onset of zygotic transcription, which involves the concomitant degradation of maternal mRNAs when the zygotic genome begins to be expressed [26,42,103].

Alternatively, these transcriptomic responses to increasing temperature could signify a more specific molecular response to heat stress that is distinct from the transcriptomic patterns of the MZT. The most robust changes in gene expression were among transcripts that encode molecular chaperones, and the induction of heat shock genes does not take place during the MZT [107,108]. Meanwhile, the decrease in abundance of thousands of maternal transcripts could be a component of the heat shock response itself, which characteristically involves the downregulation of thousands of genes [7–9,16]. In the canonical model of the heat shock response, the expression of molecular chaperones increases, but the expression of thousands of other genes decreases [1]. This reduction in gene expression serves to halt the production of proteins that could unfold, denature and cause further risk to the cell [12]. Thus, our observed transcriptional responses to heat stress suggest a strong heat shock response in early *D. melanogaster* embryos that is distinct from the transcriptional characteristics of the MZT; however, we cannot rule out the possibility of an early shift in the MZT based on these data alone.

## 5. Conclusion

Our results demonstrate that the differential regulation of relatively few genes underlies embryonic heat tolerance in *D. melanogaster* and early embryos exhibit an active transcriptomic response to heat stress despite their early stage. In the context of changing environments, there is much interest in predicting responses to thermal selection [109–111]. Based on the data we present herein, we predict that early embryonic heat tolerance will respond to future selection because (i) there is standing genetic variation for this trait within and among populations, (ii) there is gene flow between low- and high-latitude populations [41,112–114], and (iii) the requisite tweaks to the transcriptome are relatively minor and thus not likely to disrupt normal developmental processes. It is important to note that the evolutionary dynamics of adaptation of early embryonic traits may be complex due to interactions between maternal and zygotic factors. Selection likely acts on both the maternal factors that are loaded into eggs, which do not directly experience the environment of the egg, and the zygotic factors that are expressed in direct response to environmental changes. To our knowledge, the extent to which interactions between maternal and zygotic factors constrain and/or facilitate adaptive responses has not been explored in the context of climate change.

## Data Availability

The raw sequence data have been uploaded to the NCBI short read archive (accession: PRJNA1103862). Embryonic survival data and R code of the data analysis are included in the electronic supplementary material [115].
